# Effect of *Indigofera oblongifolia* on the Hepatic Oxidative Status and Expression of Inflammatory and Apoptotic Genes during Blood-Stage Murine Malaria

**DOI:** 10.1155/2019/8264861

**Published:** 2019-01-29

**Authors:** Mohamed A. Dkhil, Esam M. Al-Shaebi, Saleh Al-Quraishy

**Affiliations:** ^1^Department of Zoology, College of Science, King Saud University, Riyadh, Saudi Arabia; ^2^Department of Zoology and Entomology, College of Science, Helwan University, Cairo, Egypt

## Abstract

Malaria is a dangerous disease spread across several countries. Recent studies have focused on medicinal plants to discover alternative agents to the currently used drugs for malaria treatment. Here, we investigated the potential role of *Indigofera oblongifolia* leaf extract (IE) on hepatic inflammation in mice with *Plasmodium chabaudi*-infected erythrocytes. Female C57BL/6 mice were divided into three groups. The first group served as a control noninfected group, while the second and third groups were intraperitoneally injected with 10^6^ erythrocytes parasitized by *P. chabaudi*. Mice from the third group were treated daily with a dose of 100 mg/kg of IE for 7 days. IE significantly reduced the number of leukocytes and apoptotic cells. The numbers of CD68-positive cells decreased in the livers of mice from the treatment group. Moreover, IE raised the hepatic antioxidant levels (glutathione and catalase) and reduced the levels of hepatic oxidative stress markers (malondialdehyde, nitric oxide, and reactive oxygen species). IE regulated some functions of the genes related to immune responses, including apoptotic genes (B-cell lymphoma-2, Bax, and caspase-3) and cytokine genes (interleukin-1*β* (IL-1*β*), IL-6, interferon-*γ*, and tumor necrosis factor-*α*). Therefore, IE exerts significant effects against malaria and protects the liver from injury caused by *P. chabaudi* via antioxidant and anti-inflammatory ways.

## 1. Introduction

Malaria is a dangerous disease spread across the world, especially in the developing and underdeveloped countries. Although major progress has been reported in the fight against malaria, about 3.2 billion people are at the risk of malaria infection worldwide [1]. According to the World Health Organization, more efforts have been directed to reduce malaria-induced death by 60% as well as to eliminate malaria from many countries since 2000 [1].

The liver is the first site of sporozoite development before infection of erythrocytes [2]. The sporozoites pass from the salivary glands of the mosquitoes to the host and are transmitted to the blood stream from the liver sinusoid. In the sinusoid, sporozoites cross the sinusoidal cell layer to infect hepatocytes and grow and develop into erythrocyte-invasive forms [3]. Several studies have investigated the effector role of the liver during blood-stage malaria [4–6]. Malaria infection is associated with both acquired immune [7, 8] and innate immune [9] responses, characterized with early and intense proinflammatory cytokine-mediated effector mechanisms that kill or remove parasite-infected cells [10].

Malaria induced by *Plasmodium* exhibits resistance to drugs [11]. The increase in the prevention and control measures adopted since 2010 has reduced the mortality rates associated with malaria by 29% [12]. In malaria-endemic areas, medicinal plants have been used for treatment [13]. Plant extracts have been shown to play an important role in the treatment of malaria, owing to the presence of active components against the malarial parasite [14].

*Indigofera oblongifolia*, a traditional plant used for medicinal purposes, is known for its analgesic and anti-inflammatory effects [15]. *I. oblongifolia*, called as *hasr* in Arabic, belongs to the family Fabaceae and is prominent in Asia and Africa [15]. *I. oblongifolia* leaf extract (IE) contains polyphenols, flavonoids, and organic acids [16]. IE showed antimalarial and antioxidant activities and provides protection to the spleen from the parasite *P. chabaudi* in mice [4–6]. In the present study, we demonstrate the role of IE in the modulation of cytokine expression and apoptosis in mouse liver infected with blood-stage malaria.

## 2. Materials and Methods

### 2.1. Preparation of the Extract

We collected fresh leaves of *I. oblongifolia* from Jazan, Saudi Arabia, in March 2018. The identity of this species was confirmed at the herbarium of King Saud University (code: 9028). Leaves were air dried at 40°C for 3 hours and ground into powder. The powder was incubated in 70% methanol at 4°C for 24 h. The extract was filtered and evaporated using an evaporator machine (Heidolph, Germany). In this experiment, distilled water was used to dissolve the powder [17].

### 2.2. Determination of Phenolics and Flavonoids in the Extract

Total phenolics and flavonoids were determined according to the method described by Kim et al. [18] and Dewanto et al. [19], respectively. Gallic acid was used as the standard for total phenolics, while quercetin was used as the standard for total flavonoids.

### 2.3. Experimental Animals

Adult 10- to 12-week-old female C57BL/6 mice were used as experimental animals. Mice were fed with a standard diet and water ad libitum. All experiments were approved by the state authorities and followed Saudi Arabian rules on animal protection.

### 2.4. Infection

Animals from the noninfected control group (8 mice) were orally inoculated with distilled water. The second and third groups of mice were intraperitoneally infected with 10^6^ erythrocyte parasitized by *Plasmodium chabaudi*. The third group of mice was orally administered with 100 mg/kg of IE once daily for 7 days [4]. We chose this dose (100 mg/kg) based on our previous studies [4].

The percentage of parasitemia was determined in mice from groups 2 and 3 by Giemsa-stained blood smears collected from the tail vein [20].

### 2.5. Blood Collection

Blood was collected into heparinized tubes. Total leucocytes were counted using an automatic hematology analyzer HM5 (VetScan, USA).

### 2.6. Liver Samples

Seven days post infection, all the experimental mice were sacrificed. Liver tissues were excised and cut into small pieces.

For histological and immunohistochemical studies, liver tissues were fixed in 10% buffered formalin. For studying the antioxidant activity of IE, tissues were stored at −80°C. For gene expression experiment, liver tissues were stored in RNAlater (QIAGEN, Hilden, Germany).

### 2.7. Apoptosis Detection

The paraffin-embedded liver sections were assayed using the terminal deoxynucleotidyl transferase dUTP nick end labeling (TUNEL) Apoptosis Kit (GenScript, Piscataway, NJ, USA) according to the manufacturer's protocol [21].

### 2.8. Immunohistochemical Detection of CD68 Expression

Immunohistochemical detection of CD68-positive cells in liver sections was carried out. In brief, the deparaffinized sections were incubated in 10 mM citrate buffer solution for antigen retrieval, followed by the treatment of sections with 3% hydrogen peroxide (H_2_O_2_) in phosphate buffer solution for 5 min. Liver sections were blocked with 10% normal goat serum for 10 min at 37°C and overnight incubated at 4°C with equal amounts of normal goat IgG as a negative control. The avidin biotin affinity system was used to treat the liver sections, followed by washing and staining of the sections with the 3,3′-diaminobenzidine substrate.

### 2.9. Phagocytic Activity

Five mice from each group were intravenously injected with phosphate buffer containing 2.9 × 10^8^ of green fluorescent beads (Duke Scientific, Palo Alto, CA, USA). Seven minutes later, all mice were sacrificed. Liver cryosections were prepared, and fluorescence intensity was determined using ImageJ software [22] after examination under an Olympus fluorescent microscope [23].

### 2.10. Antioxidant Activity in the Liver

Mouse liver tissues were homogenized in an ice-cold medium containing 300 mM sucrose and 50 mM Tris-HCl [24]. Centrifugation was carried out at 500 × *g* for 10 min at 4°C. The supernatant (10%) was used for different biochemical estimations.

Glutathione (GSH) level in the liver homogenate was determined according to the method described by Ellman [25]. The concentration of nitrite oxide (NO) in the liver homogenate was assayed according to the method described by Berkels et al. [26]. For evaluation of the lipid peroxidation level, the method described by Ohkawa et al. [27] was used. Catalase activity was determined according to the method described by Aebi [28].

### 2.11. Gene Expression

Total RNA from mouse liver was isolated using TRIzol (QIAGEN, Hilden, Germany). RNA was quantified using the ND-1000 spectrophotometer (NanoDrop Technologies, Wilmington, DE, USA) [22]. To process RNA for real-time quantitative polymerase chain reaction (RT-qPCR), we treated samples with DNase (Applied Biosystems, Darmstadt, Germany). Samples were then converted into cDNA using the reverse transcription kit (QIAGEN, Hilden, Germany). To carry out PCR, the ABI Prism 7500HT sequence detection system (Applied Biosystems, Darmstadt, Germany) with SYBR green PCR master mix from QIAGEN (Hilden, Germany) was used. The following genes were investigated: interleukin-1*β* (IL-1*β*) (Mm_Il1b_2_SG, cat. no. QT01048355), interleukin-6 (IL-6) (Mm_Il6_1_SG, cat. no. QT00138663), interferon gamma (INF-*γ*) (Mm_Ifng_1_SG, cat. no. QT01038821), tumor necrosis factor-alpha (TNF-*α*) (Mm_Tnf_1_SG, cat. no. QT00104006), Bcl2-associated X protein (Bax) (Mm_Bax_1_SG, cat. no. QT00102536), B-cell lymphoma 2 (Bcl2) (Mm_Bcl2_3_SG, cat. no. QT00156282), and caspase-3 (Casp3) (Mm_ Casp3_1_SG, cat. no. QT00260169). The used primers were purchased from QIAGEN. PCR reaction was performed as described by Dkhil et al. [4]. The 2^−ΔΔ^CT method was used to determine the fold change in mRNA expression [29].

### 2.12. Statistical Analysis

Statistical comparison among the studied groups was carried out using one-way analysis of variance (ANOVA). Duncan's *t*-test and a statistical package program (SPSS version 17.0) were used. The statistical significance for all data was set at *p* ≤ 0.01.

## 3. Results

The concentration of phenolic compounds present in IE (gallic acid equivalent) was 77 ± 4 *μ*g/mg, while the flavonoid content (quercetin equivalent) was 25 ± 3 *μ*g/mg.

**Figure 1:**
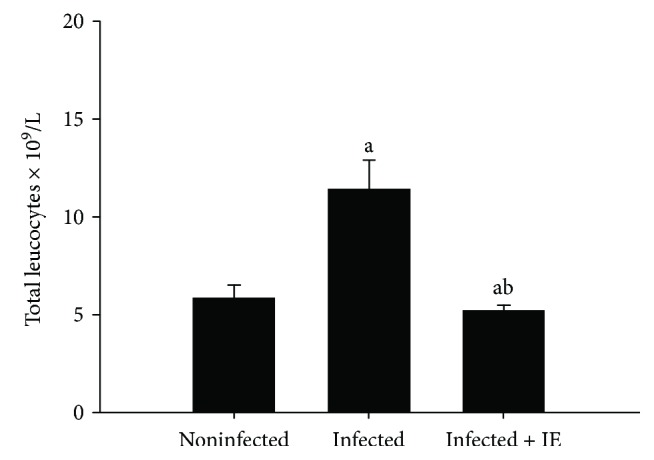
IE-induced changes in mouse leucocytes infected with *P. chabaudi*. Data are expressed as means ± SD; a: significant change at *p* ≤ 0.05 with respect to the control group; b: significant change at *p* ≤ 0.05 with respect to the infected (−IE) group.

The infection induced by *P. chabaudi* in female C57L/B6 mice causes an increase in leucocyte count; however, treatment with IE resulted in a significant difference in the leucocyte count (*p* < 0.05) (Figure 1).

**Figure 2:**
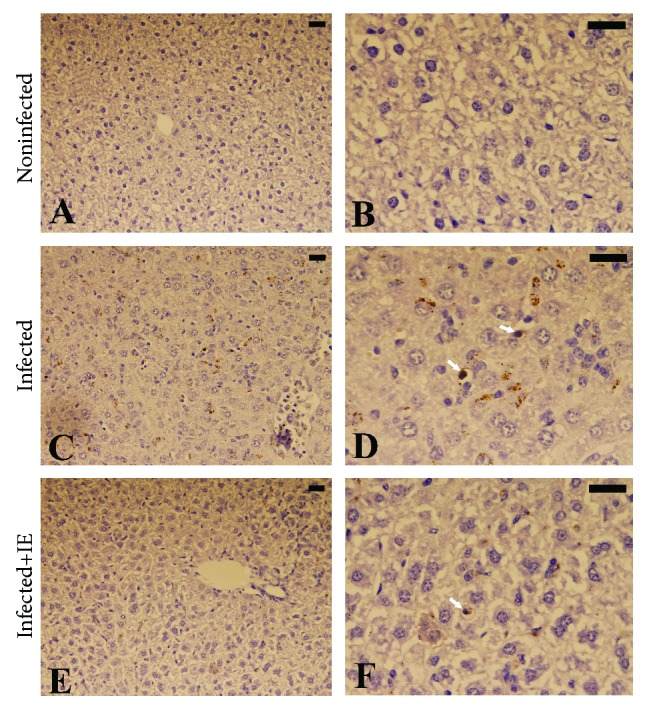
Apoptotic changes in mouse livers infected with *P. chabaudi* and treated with IE. (a, b) Control liver. (c, d) Infected liver with TUNEL apoptotic cells (white arrow) and hemozoin. (e, f) Liver from the treatment group with a few TUNEL-positive apoptotic cells. Scale bar = 25 *μ*m.

**Figure 3:**
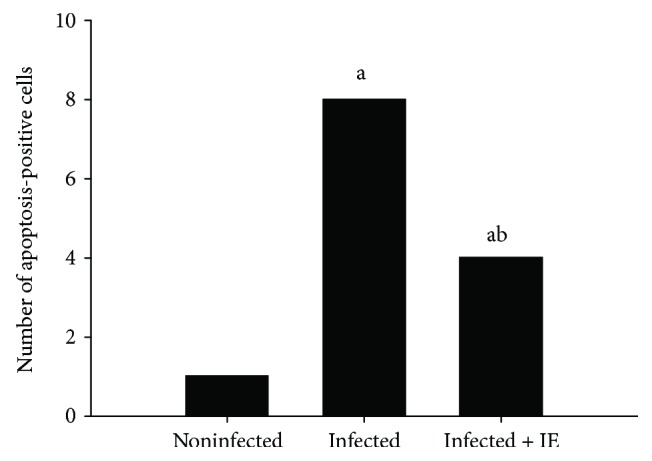
IE-induced changes in the number of apoptotic cells in the liver of mice infected with *P. chabaudi* on day 7. Data are expressed as means ± SD; a: significant change at *p* ≤ 0.05 with respect to the control group; b: significant change at *p* ≤ 0.05 with respect to the infected (−IE) group.

Microscopic examination of liver sections following TUNEL staining (Figure 2) revealed the increase in the number of apoptotic cells in the samples from the infected group. On the contrary, the number of TUNEL-positive cells decreased by about 50% in the samples from the treatment group (Figure 3).

**Figure 4:**
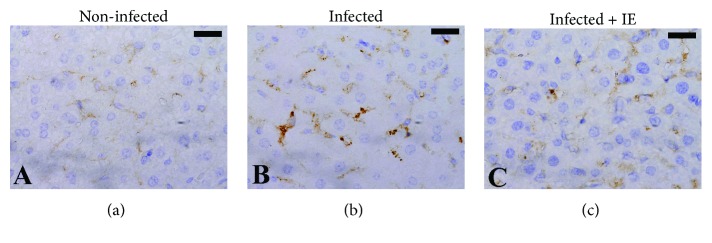
Liver section labeled with the macrophage detection anti-CD68 antibody from mouse infected with *Plasmodium chabaudi*. (a) Control mouse, (b) mouse infected with *P. chabaudi*, and (c) mouse infected with *P. chabaudi* and treated with IE. CD68-positive cells appeared brown. Scale bar = 25 *μ*m.

**Figure 5:**
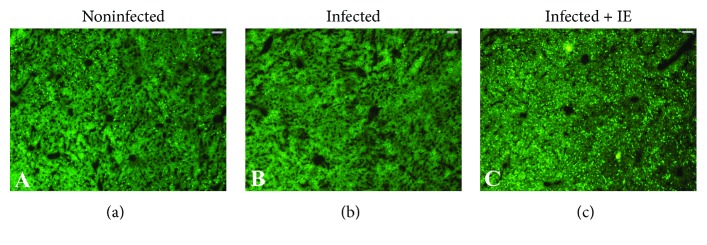
Phagocytic activity in the mouse liver. (a) Control liver with more fluorescent particles. (b) *P. chabaudi*-infected liver with reduced number of fluorescent particles. (c) IE-treated liver with a strong fluorescence signal. Bar = 25 *μ*m.

**Figure 6:**
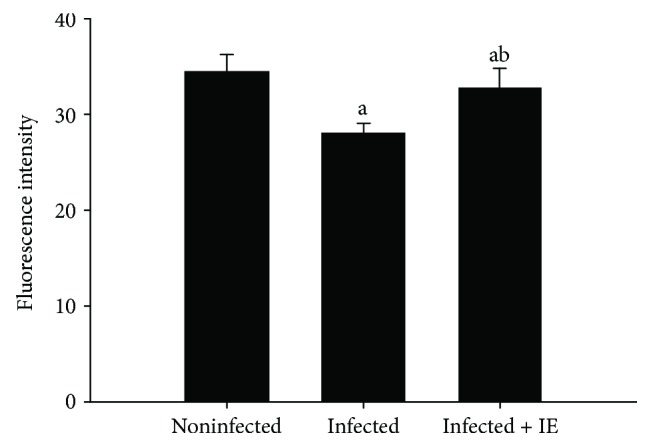
Semiquantitative evaluation of the fluorescence intensity of five cryosections per mouse. Data are expressed as means ± SD; a: significant change at *p* ≤ 0.05 with respect to the control group; b: significant change at *p* ≤ 0.05 with respect to the infected (−IE) group.

To evaluate the phagocytic activity during infection and after treatment of the infected mice with IE, we examined the number of CD68-positive cells in liver sections (Figure 4). IE treatment significantly decreased the number of CD68-positive cells in *P. chabaudi*-infected liver samples (Figure 4). The intensity of fluorescent particles as an indicator of phagocytic activity reduced in the liver sections from the infected group (Figure 5) but significantly increased in the liver sections from the IE-treated group (Figures 5 and 6).

Biochemical analysis of mouse liver samples revealed a marked increase in the expression level of NO and MDA by approximately 89.91% and 53.33%, respectively, and a significant decrease (*p* ≤ 0.05) in the level of GSH and catalase activity (Table 1). IE treatment significantly reduced the infection-induced increase in NO and MDA levels and increased the level of GSH as well as the activity of catalase (Table 1).

**Figure 7:**
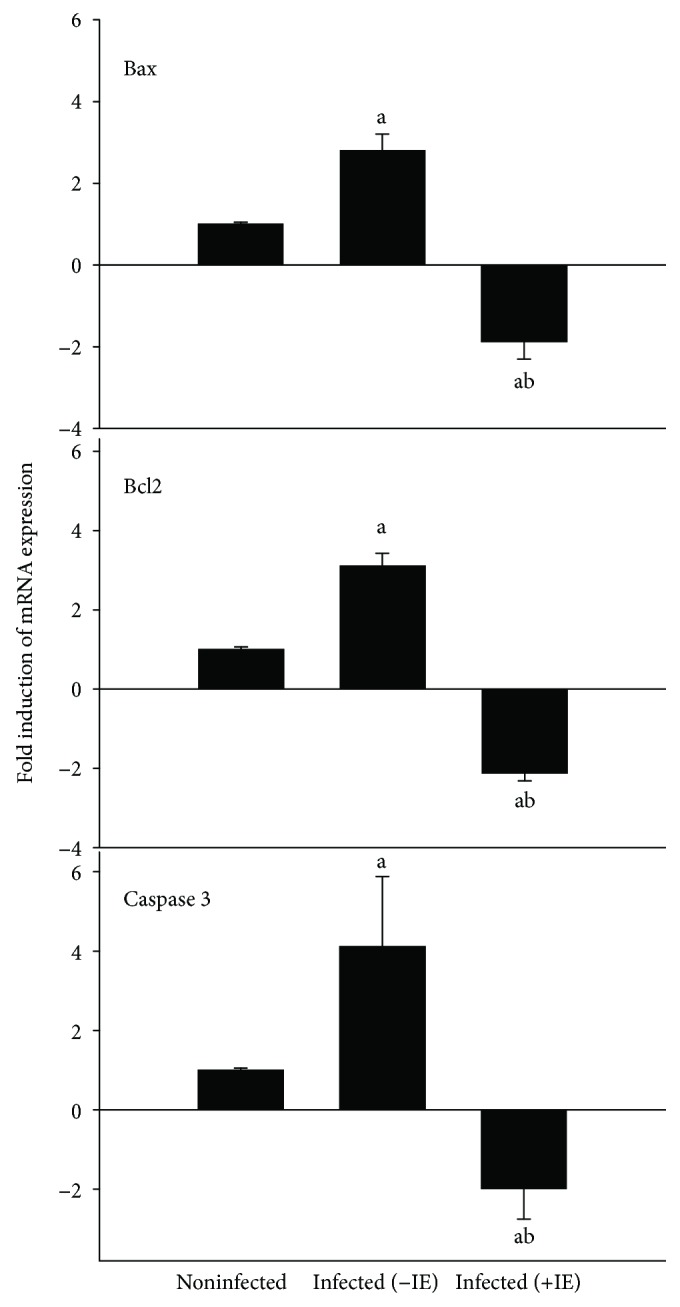
IE-induced changes in the expression level of apoptotic genes (Bcl2, Bax, and caspase-3) in the liver of mice infected with *P. chabaudi*. Data are expressed as means ± SD; a: significant change at *p* ≤ 0.05 with respect to the control group; b: significant change at *p* ≤ 0.05 with respect to the infected (−IE) group.

*P. chabaudi*-infected mice showed a significant upregulation in the mRNA expression of Bax, Bcl2, and Casp3 in liver tissues as compared to the noninfected mice (Figure 7). In contrast, a significant decrease in the mRNA levels of Bax, Bcl2, and Casp3 was observed in the mice following IE treatment (Figure 7).

**Figure 8:**
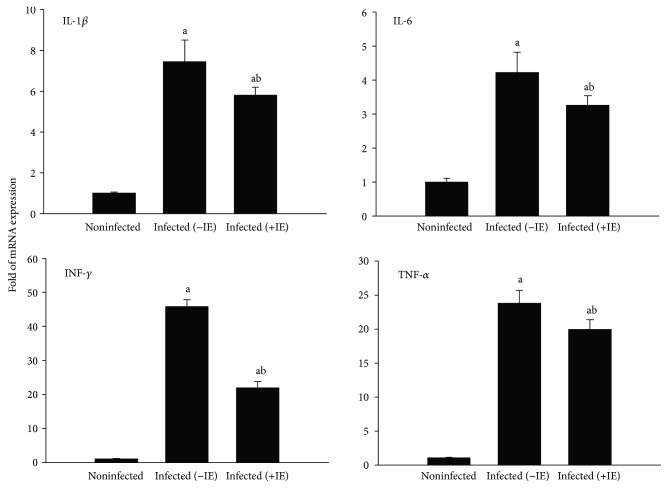
IE-induced changes in the expression levels of hepatic IL-1*β*, IL-6, IFN-*γ*, and TNF-*α* genes in the mice infected with *P. chabaudi* on day 7. Data are expressed as means ± SD; a: significant change at *p* ≤ 0.05 with respect to the control group; b: significant change at *p* ≤ 0.05 with respect to the infected (−IE) group.

We observed that the infection induced upregulation in the mRNA expression of liver IL-1*β*, IL-6, IFN-*γ*, and TNF-*α*, while treatment of *P. chabaudi*-infected mice with IE resulted in a significant downregulation in the expression levels of these cytokines (Figure 8).

## 4. Discussion

Malaria remains as one of the dangerous diseases affecting people in several countries. IE reduces the induced parasitemia following *P. chabaudi* infection [4, 5, 30], owing mainly to the presence of an active antimalarial component, quinine [31].

Frevert and Nardin [2] reported that the liver is the first site of preerythrocytic development of *Plasmodium*. These authors documented that the liver serves as an effector against blood-stage malaria [2], wherein the liver endothelial system eliminates the parasitized erythrocytes possibly by phagocytosis [32]. Savil and Fadok [33] reported membrane changes in response to apoptosis that resulted in the formation of apoptotic bodies, which could be easily phagocytosed by macrophages. In the hepatic tissue, Kupffer cells comprise about 70-80% of the liver macrophages and may phagocytize the parasitized erythrocytes as well as hemozoin granules [34]. IE treatment increased the number of phagocytic cells and reduced *P. chabaudi*-induced parasitemia via phagocytosis.

Oxidative stress due to infection led to the activation of molecular pathways that drive inflammation and could directly induce tissue injury. In our previous work, we reported only that IE may effectively improve the liver histopathological changes associated with *P. chabaudi* infection [30]. Here, we reported for the first time that the induced pathological changes in the liver are due to parasite-induced oxidative stress that may cause cell damage and subsequently lead to cell death. In addition, IE ameliorated the apoptotic changes induced in response to the parasite infection.

In this study, we observed an increase in lipid peroxidation and depletion in the GSH level accompanied with the inhibition of antioxidant enzyme activities following malarial infection. Lipid oxidation results in cell membrane damage and loss of plasma membrane integrity, thereby causing cell death. Superoxide dismutase has a major role in scavenging O_2_ and may react with NO as an antioxidant. In addition, catalase degrades hydrogen peroxide (H_2_O_2_) into H_2_O and O_2_ and causes reduction in the level of ROS. Several studies have highlighted the antioxidant role of IE especially in the spleen and kidney [5, 6, 17]. In this study, the hepatic GSH level and catalase activity were significantly reduced upon infection, while treatment with IE resulted in the restoration of the GSH level and catalase activity. However, NO and MDA levels decreased after treatment with IE.

The liver is responsible for the systemic response to blood-stage malaria and produces cytokines, which are important in local immune response [35]. The number of leucocytes increased (Figure 1) in response to injuries caused by *P. chabaudi* [33]. IE is a natural product with anti-inflammatory, antioxidant, antimalarial, and hepatoprotective effects [5, 30]. Previous studies have documented that *Indigofera oblongifolia* contains active compounds such as flavonoids and alkaloids as well as the antimalarial compound, quinine [16]. The antioxidant and anti-inflammatory effects of IE were reported in rat liver with severe lead acetate-induced inflammation [16].

## 5. Conclusions

The present study highlights the antioxidant, anti-inflammatory, and antiapoptotic effects of IE in *P. chabaudi*-induced hepatic injury, as evident from the effects of IE on hepatic oxidative damage, regulation of inflammatory cytokines, and apoptotic gene expression.

## Figures and Tables

**Table 1:** Effect of IE on the level of hepatic glutathione (GSH), catalase (CAT), malondialdehyde (MDA), and nitric oxide (NO) in mice infected with *P. chabaudi*.

Groups	GSH (mg/kg)	CAT (U/g)	MDA (nmol/g)	NO (*μ*mol/g)
Noninfected	11.8 ± 3.2	8.3 ± 0.9	4.1 ± 0.4	165.3 ± 17
Infected (−IE)	4.9 ± 0.1^a^	5.4 ± 0.2^a^	6.2 ± 0.2^a^	313.9 ± 13.8^a^
Infected (+IE)	11.6 ± 1.1^ab^	18.1 ± 2.3^ab^	3.9 ± 0.6^b^	168.8 ± 1.1^b^

Values are means ± SD. ^a^Significant against the noninfected group at *p* ≤ 0.05; ^b^Significant against the infected (−IE) group at *p* ≤ 0.05, (*n* = 8).

## Data Availability

The data set supporting our results is included within the article.

## Abbreviations

Bax: Bcl2-associated X protein
Bcl2: B-cell lymphoma 2
Casp3: Caspase-3
IE: *Indigofera oblongifolia* leaf extract
IL-1*β*: Interleukin-1*β*
IL-6: Interleukin-6
INF-*γ*: Interferon gamma
MDA: Malondialdehyde
NO: Nitric oxide
RT-qPCR: Real-time quantitative polymerase chain reaction
TNF-*α*: Tumor necrosis factor-alpha
TUNEL: Terminal deoxynucleotidyl transferase dUTP nick end labeling.
